# Establishment of a Rat Model of Portal Vein Ligation Combined with *In Situ* Splitting

**DOI:** 10.1371/journal.pone.0105511

**Published:** 2014-08-21

**Authors:** Libin Yao, Chonghui Li, Xinlan Ge, Hongdong Wang, Kesen Xu, Aiqun Zhang, Jiahong Dong

**Affiliations:** 1 Department of Hepatobiliary Surgery, Qilu Hospital of Shandong University, Jinan, Shandong Province, China; 2 Hospital and Institute of Hepatobiliary Surgery, Chinese PLA General Hospital, Beijing, China; St. Luc University Hospital, Belgium

## Abstract

**Background:**

Portal vein ligation (PVL) combined with in situ splitting (ISS) has been shown to induce remarkable liver regeneration in patients. The purpose of this study was to establish a model of PVL+ISS in rats for exploring the possible mechanisms of liver regeneration using these techniques.

**Materials and Methods:**

Rats were randomly assigned to three experimental groups: selective PVL, selective PVL+ISS and sham operation. The hepatic regeneration rate (HRR), Ki-67, liver biochemical determinations and histopathology were assessed at 24, 48, and 72 h and 7 days after the operation. The microcirculation of the median lobes before and after ISS was examined by laser speckle contrast imaging. Meanwhile, cytokines such as TNF-α, IL-6, HGF and HSP70 in regenerating liver lobes at 24 h was investigated by RT-PCR and ELISA.

**Results:**

The HRR of PVL+ISS was much higher than that of the PVL at 72 h and 7 days after surgery (p<0.01). The expression of Ki-67 in hepatocytes in the regenerating liver lobe was stronger in the PVL+ISS group than in the PVL group at 48 and 72 h (p<0.01). There was a significant reduction in microcirculation blood perfusion of the left median lobe before and after ISS. Liver biochemical determinations and histopathology demonstrated more severe hepatocyte injury in the PVL+ISS group. Both the mRNA levels of TNF-α and IL-6 and the protein levels of TNF-α, IL-6 and HGF in regenerating liver lobes were higher in the PVL+ISS than the PVL alone.

**Conclusions:**

The higher HRR in the PVL+ISS compared with the PVL confirmed that we had successfully established a PVL+ISS model in rats. The possible mechanisms included the reduced microcirculation blood perfusion of the left median lobe and up-regulation of cytokines in the regenerating lobes after ISS.

## Introduction

Hepatectomy is the only potentially curative therapeutic option for many patients with primary or secondary liver tumors [1]. However, insufficient remnant liver restricts its application [2]. Many methods have been introduced to increase the remnant liver volume owing to the unique regenerative capacity of the liver. In the 1980s, Makuuchi first applied selective portal vein embolization to induce hypertrophy of the remnant liver in patients requiring extended hepatectomy because of large or multiple liver tumors with the aim of avoiding postoperative liver failure [3]. Since then, the application of selective portal vein embolization (PVE) or selective portal vein ligation (PVL) has increased among hepatobiliary surgeons as an important option for patients who cannot tolerate hepatectomies because of insufficient remnant liver volume. Recent studies have shown that embolizing or ligating the portal vein of the lobes planned for resection could increase the future remnant liver volume 8% to 46% between 2 and 8 weeks [4]–[6]. That is to say, sufficient hypertrophy of the remnant liver is not always achieved using PVE or PVL.

In 2012, Schnitzbauer AA et al. [7] published an article titled “Right Portal Vein Ligation Combined With In Situ Splitting Induces Rapid Left Lateral Liver Lobe Hypertrophy Enabling 2-Staged Extended Right Hepatic Resection in Small-for-Size Settings” in the Annals of Surgery. They introduced a new strategy of 2-staged extended right hepatic resection, which was a right PVL combined with in situ splitting along the falciform ligament in initial surgical exploration to induce hypertrophy of the left lateral lobe in patients with marginally resectable or nonresectable primary and secondary liver tumors. This new strategy induced a 74% median volume increase in the left lateral lobe in a mean of 9 days, which is impossible to achieve by PVE or PVL. de Santibanes and Clavien [8] named this technique “Associating Liver Partition and Portal vein ligation for Staged hepatectomy”, or ALPPS. This technique has stimulated great interest in the field of hepatobiliary surgery and is thought to open a new chapter in the history of liver surgery [9]–[11].

However, the exact mechanisms of the spectacular regenerative response in PVL+ISS are not clear, and this method also has some drawbacks such as a high incidence of morbidity and mortality. In the present study, we established a model of PVL+ISS in rats to explore the possible mechanisms of the spectacular regenerative response in PVL+ISS. This research will open a door for further experimental studies of PVL+ISS-related events.

## Materials and Methods

### Ethics statement

All animal treatments were strictly in accordance with the international ethical guidelines and the National Institutes of Health Guide concerning the Care and Use of Laboratory Animals. This experiment was carried out strictly in accordance with the ARRIVE guidelines for animal research [12]. The experimental procedures were conducted according to the Guide for the Care and Use of Laboratory Animals and were approved by the Institutional Animal Care and Use Committee of the Chinese PLA General Hospital (Protocol Number: 2014-X9-01). The animals were treated humanely and protected animal welfare. After surgical procedure, the animals recovered in a warm environment with free access to water and food and were monitored every 12 hours. Every effort was made to minimize any suffering of the animals. All the animals were sacrificed using chloral hydrate to obtain the specimens at each time point.

### Animals

The experiments were performed on male Sprague-Dawley rats weighing 250–280 g (The Experimental Animal Center, The Academy of Military Medical Sciences, Beijing, China). The animals were housed under specific pathogen-free conditions with a 12-h light/dark cycle and permitted ad libitum access to standard rodent chow and water.

### Experimental Design and Operative Procedures

All animals were randomly assigned to one of three experimental groups: selective PVL, selective PVL combined with in situ splitting (PVL+ISS) and sham operation (SHAM). The rats fasted 12 h before the operation and were anesthetized with ether inhalation. All operations were performed under an operating microscope (Binocular Operation Microscope; Type GX.SS.22-3; Shanghai Medical Optical Instruments Co, Ltd. China). The liver was freed from its ligaments after a midline laparotomy. In sham-operated animals, the hepatic artery, portal vein and bile duct were dissected without ligation, and then the abdomen was closed by a double running suture.

Portal Vein Ligation: Selective PVL was performed on the caudal lobe, left lateral and left median lobes, and the right lobe. The right median lobe was preserved to regenerate. After careful dissection of the hepatic artery and bile duct, the corresponding portal veins of the lobes were suture ligated with 6-0 silk.

In Situ Splitting: As we known, differently from humans the liver of rat is composed of relative independent lobes such as left lobe, median lobe, right lobe and caudate lobe. The reason for choosing the median lobe to split the liver is that the shape and anatomy of the median lobe are similar to humans’ livers because the median lobe is the only one with two branches of the portal vein, none of other lobes get the same blood supply architecture. Therefore the ischemic line can emerge immediately at the right side of the falciform ligament when one of portal vein branches to median lobe was ligated. The right median lobe was chosen as non-ischemia remnant due to completely independent blood supply from the portal vein and relatively smaller size with easier reflect regenerating ability.

**Figure 1:**
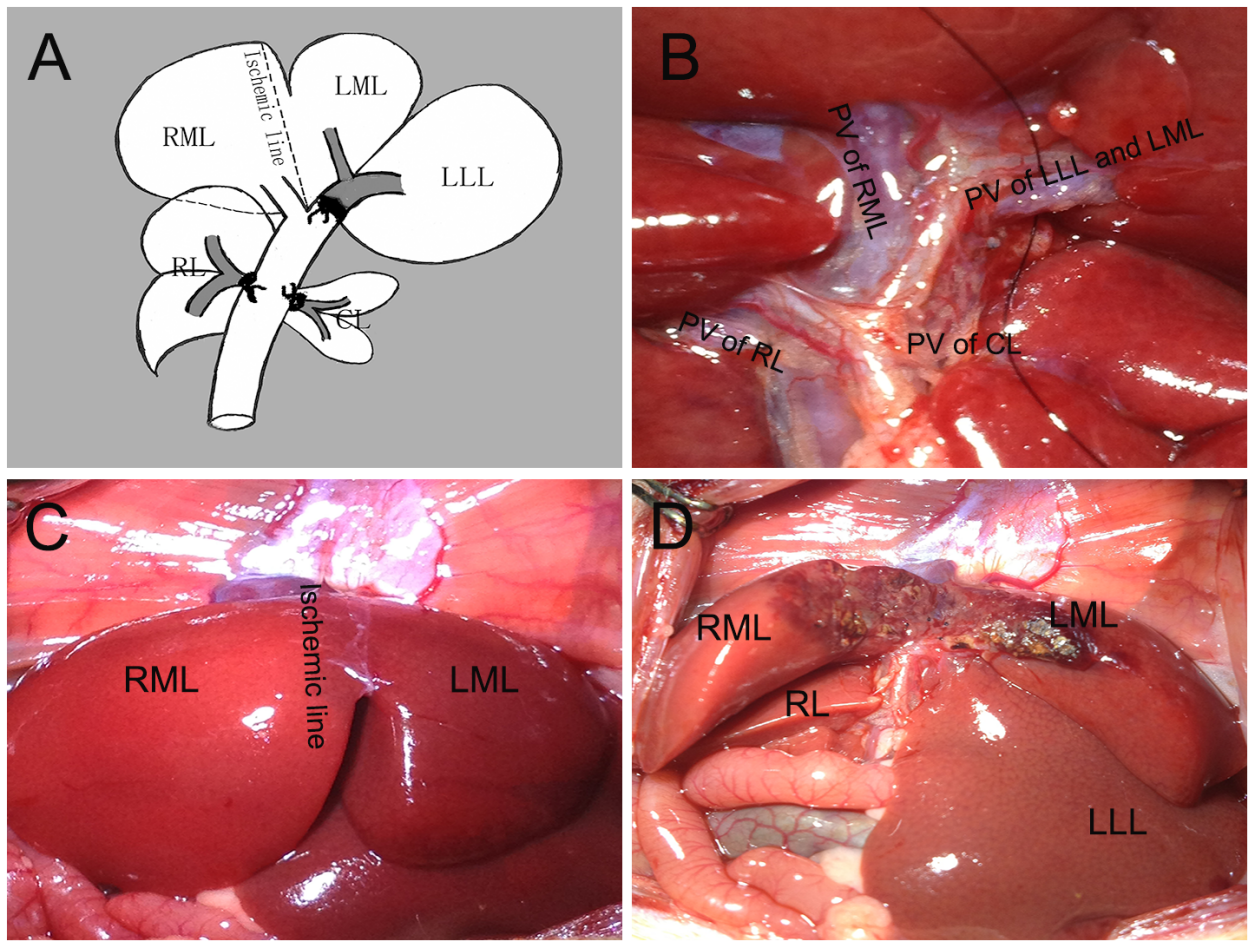
Illustrations of surgical procedure of PVL+ISS. (**A**) Schematic anatomy of liver lobes and portal veins: right median lobe (RML), left median lobe (LML), left lateral lobe (LLL), right lobe (RL), caudate lobe (CL). The silk knots represent the ligated lobes. (**B**) Portal veins (PV) of rats in surgery. (**C**) Ischemic line emerged to the right of the falciform ligament when the left branch of the portal vein was ligated. (**D**) The median lobe was in situ split along the ischemic line.

The in situ splitting was performed along the border between the left median lobe and the right median lobe. When the common trunk of the portal vein of the left lateral and left median lobe was ligated, the ischemic line (i.e., the border) emerged immediately at the right side of the falciform ligament. Then, total liver parenchymal transection was performed along the ischemic line. Briefly, a microscopic tweezers was used to clamp the liver parenchyma along the ischemic line. Then, the observed avascular zones of the liver parenchyma along the ischemic line were gradually perforated using the microscopic tweezers cutting the small parts of the liver parenchyma after ligating the left and right sections with an 8-0 silk suture until the entire liver parenchyma was completely transected. Some small vascular traffic branches were often encountered during splitting liver parenchyma. Electrocoagulation or suture ligature of 8-0 silk was used for hematischesis to hemorrhage of these communicating branches of small vessels in the transection surface. Next, the transection surface of the left median lobe was wrapped with a small piece of sterile plastic film to prevent adhesion to the two transection surfaces. The abdomen was closed with a 4-0 silk double layer running suture at the end of surgery **(** **figure 1** **)**. There were no death and serious complications occurred during operation or observation period (7 days).

The animals of each group were sacrificed at 24, 48, and 72 h and 7 days after the operation (n = 6 for each time point). The blood samples collected from the inferior vena cava at different time points were centrifuged at 3000 *g* for 10 min, and the serum was stored at −80°C until the analyses for liver biochemical determinations were performed. The total liver was removed and divided into the right median lobe, left median lobe, left lateral lobe, right lobe and caudal lobe. After being weighed, approximately 200 mg of liver tissue from the right and left median lobes was immediately frozen in liquid nitrogen and then stored at −80°C. The remnant liver lobes were fixed in 10% formaldehyde.

### Laser Speckle Contrast Imaging

Laser speckle contrast imaging (LSCI) can noninvasively map the surface blood flow of tissues [13], [14]. We randomly selected six rats in the PVL+ISS group to compare the microcirculation blood perfusion of the right median and left median lobes before and after ISS. Briefly, the animals underwent ether anesthesia in the prone position. LSCI was performed 5 min after laparotomy, PVL and PVL+ISS in identical rats with the FLPI-2 (Full-Field Laser Perfusion Imager; Moor Instruments, Essex, UK) in the high resolution/high speed setting at a display rate of 25 Hz, time constant of 1.0 s and camera exposure time of 20 ms. The scanning distance was set at 20 cm, and the LSCI was performed in an operating room with a constant temperature of 26°C. The LSCI measurements of the right median and left median lobes were performed at the following time points: 5 min after laparotomy as a baseline, 5 min after PVL and 5 min after PVL+ISS. The values of liver microcirculation perfusion were expressed as percentages of the baseline values, namely PVL% and PVL+ISS%.

### Hepatic Regeneration Rate

The hepatic regeneration rate (HRR) of the right median lobe was calculated with the following formula:
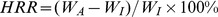
Where W_A_ represents the actual weight of the right median lobe measured using a laboratory micro scale at each sacrificed time point. W_I_ represents the initial right median lobe weight before the operative procedure, which was calculated by the weight of the rat ×0.74% (0.74% was the mean percent of the liver weight of the right median lobe to the body weight of 15 normal male Sprague-Dawley rats weighing 250–280 g).

### Hepatocellular Damage and Hepatic Synthetic Function

The collected serum was analyzed for alanine aminotransferase (ALT), aspartate aminotransferase (AST), albumin (ALB) and total bilirubin (TBIL) in the Clinical Biochemistry Department using a serum multiple biochemical analyzer (Cobas-Mira Plus, Roche, Manheim, Germany).

### Histological Examination

The liver tissues were immersion fixed in 10% formaldehyde, embedded, sectioned, and stained with hematoxylin-eosin (H–E). The liver sections of the right median lobes were immunostained for Ki-67 (mouse monoclonal antibody Ki-67, BD Biosciences, USA) according to the manufacturer’s instructions. All immunostains were counterstained with hematoxylin. The number of Ki-67-positive hepatocytes was determined in 5 random visual fields (200×). The necrotic areas of left median lobes were quantified in 10 random visual fields (100×) using Adobe Photoshop CS 5. Necrosis was expressed as the percentage of necrotic tissue: 0, no necrosis; 1, less than 25%; 2, 25%–50%; 3, 50%–75%; and 4, at least 75% necrosis [15]. All histologic analyses were performed in a blinded fashion with respect to the experimental groups.

### Quantitative Real-Time Polymerase Chain Reaction

The total RNA was extracted from liver tissue using TRIzol reagent (Invitrogen Life Technologies, Carlsbad, CA, USA). After being spectrophotometrically quantified, 4 µg of total RNA was reverse-transcribed into cDNA using the RevertAid First Strand cDNA Synthesis Kit and Oligo-dT primers (Thermo Fisher Scientific Ins, Burlington, ON, Canada). Specific primers were designed for tumor necrosis factor-α (TNF-α), interleukin-6 (IL-6), hepatocyte growth factor (HGF) and heat shock protein 70 (HSP70). Rat glyceraldehyde-3-phosphate dehydrogenase (GAPDH) was employed as an endogenous control **(** **Table 1** **)**. The quantitative real-time PCR amplification was performed with SYBR-Green PCR Master Mix (TakaRa Bio, Inc, Dalian, China) using an iCycler iQ2 RT-PCR Detection system (Bio-Rad, USA). The conditions of PCR amplification were carried out as follows: 1 cycle of 95°C for 30 s; followed by 40 cycles of 95°C for 5 s, 55°C (for GAPDH, HGF and HSP70) or 58°C (for TNF-α and IL-6) for 15 s, and 72°C for 15 s. The relative mRNA expression levels were calculated using the 2^−ΔΔCt^ method [16]. The results represent an x-fold induction versus the baseline levels in the SHAM group.

**Table 1:** Primer sequences.

Symbol	Forward(5′-3′)	Reverse(5′-3′)
GAPDH	ACCACAGTCCATGCCATCAC	TCCACCACCCTGTTGCTGTA
TNF-α	AAATGGGCTCCCTCTCATCAGTTC	TCTGCTTGGTGGTTTGCTACGAC
IL-6	ACAGCGATGATGCACTGTCAG	ATGGTCTTGGTCCTTAGCCAC
HGF	TCCTGTGCCAAAACAAAACA	GGTGCTGACTGCATTTCTCA
HSP70	GGCTAGAGACAGACTCTTGATGG	CTCAGTTTGTAGGGATGCAAGG

### ELISA for Proinflammatory Cytokine and HGF Response

Regenerating liver samples were homogenized in buffer (phosphate-buffered saline solution, pH 7.4) and centrifuged (10000 *g*; 4°C; 10 min), and the supernatant was used for analysis of TNF-α, IL-6 and HGF. The concentrations of cytokines in the liver tissue were measured with an enzyme-linked immunosorbent assay (Rat TNF-α; sensitivity: 16 pg/ml, eBioscience, San Diego, USA. Rat IL-6, sensitivity: 14–36 pg/ml, and HGF; sensitivity: 1.33–12.1 pg/ml, RnD Systems, Minneapolis, USA) according to the manufacturer’s instructions. All samples were measured in duplicate in a 96-well microtiter plate. The concentrations of cytokines were calculated from the standard curve. The total protein concentration of the liver tissues was measured with a BCA Protein Assay Kit (Applygen Technologies Inc. Beijing, China), and the cytokine concentrations of the liver tissues were expressed in picograms per milligram of hepatic total protein.

### Statistics

All data are expressed as means ± standard deviation (SD). Differences between the groups were assessed by one-way analysis of variance (ANOVA, LSD post-test), Student’s *t*-test and the Mann-Whitney test. A significant difference was assumed when P was less than 0.05. Statistics were performed using SPSS, version 18.0, statistical software (SPSS Inc, Chicago, IL).

## Results

### PVL+ISS Accelerated Hepatic Regeneration

**Figure 2:**
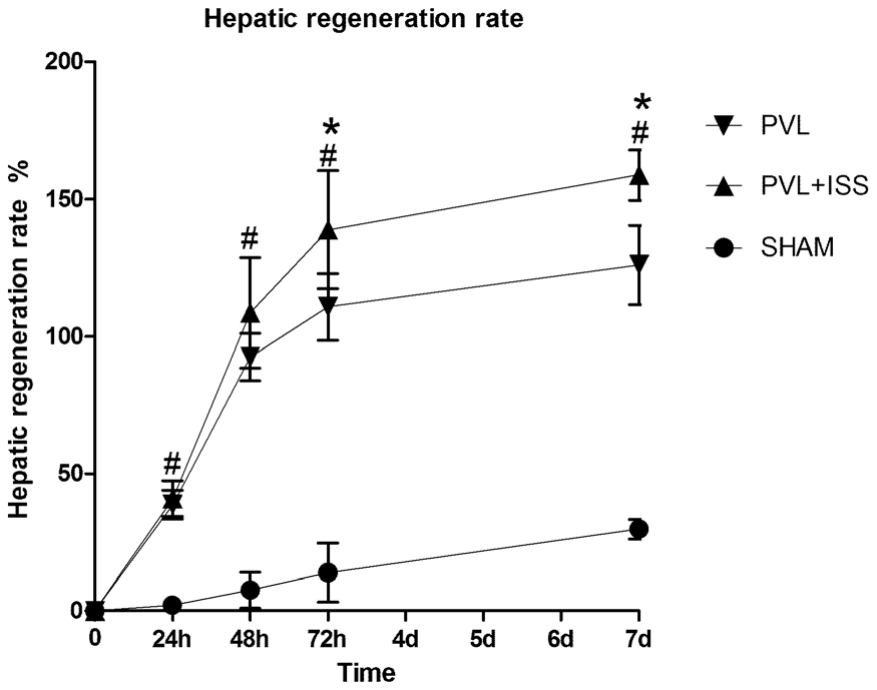
The hepatic regeneration rate (HRR) of the right median lobe after SHAM, PVL and PVL+ISS. Values are means±SD. The HRR of PVL or PVL+ISS was significantly different than the SHAM group at all time points, and the HRR of PVL+ISS was significantly different compared with the PVL group at 72 h and 7 days, but no obvious difference was observed at 24 and 48 h (^#^ *P*<0.01 compared with the sham group; *P<0.01 compared with the PVL group).

To determine the effects of PVL and PVL+ISS on liver regeneration, we measured the hepatic regeneration rate (HRR) and markers of hepatocyte proliferation of the right median lobe. The HRRs for both PVL and PVL+ISS were obviously higher than the SHAM group at all time points. Compared to the PVL group, PVL+ISS induced a greater regeneration response with an increased HRR (138.95±21.47% versus 110.71±12.21%, *p*<0.01) at 72 h and (158.92±9.17% versus 126.04±14.52%, p<0.01) at 7 days after operation. In addition, the HRR of the PVL group was lower than that of the PVL+ISS group at 48 h, although the difference was not significant (92.51±8.60% versus 108.61±20.21%). There was no significant difference between the two groups at 24 h (data not shown), **(** **Fig. 2** **)**.

**Figure 3:**
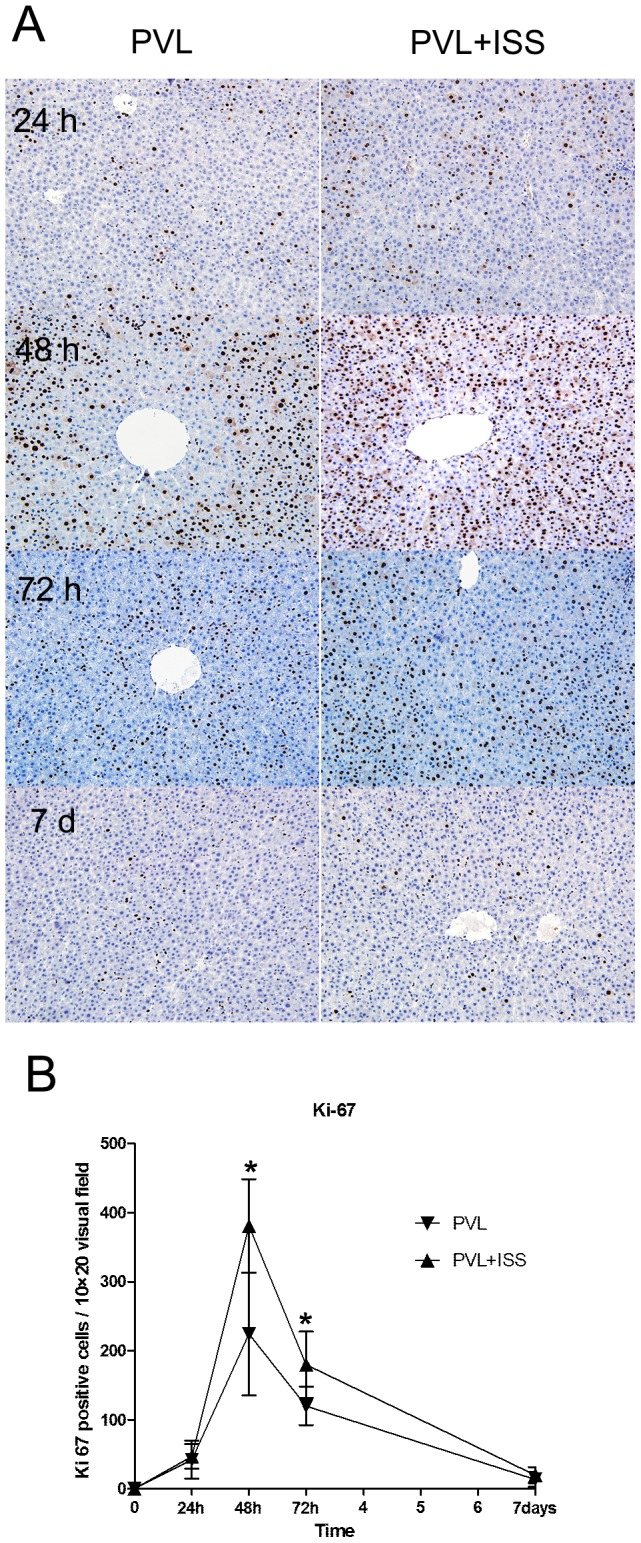
The number of Ki-67-positive hepatocytes in regenerating lobes after PVL and PVL+ISS. (**A**) Immunohistochemical staining for Ki-67 in regenerating lobes at all time points. Hepatocytes with nuclear deposition of brownish pigment were considered positive (original magnification 200×). (**B**) The regenerating lobes were analyzed for Ki-67-positive hepatocytes at all time points. The number of positive hepatocytes per visual field (200×) is presented. The increase in Ki-67-positive cells shows a significant difference at 48 and 72 h (*p<0.01 and *p<0.05, respectively), while no significant difference was observed at 24 h and 7 days.

To further explore the characteristics of the hepatic regenerative response, we assessed the expression of Ki-67, which is a nuclear antigen associated with hepatocyte proliferation **(** **Fig. 3** **)**. The number of Ki-67-positive hepatocytes per visual field in the regenerating liver lobe was greater in the PVL+ISS group than in the PVL group at 48 h (380.83±67.65 versus 224.10±88.60, p<0.01) and 72 h (179.77±48.08 versus 119.93±27.58, p<0.05). However, there was no significant difference between the two groups at 24 h (data not shown). Meanwhile, both groups showed a similar low amount of proliferating hepatocytes per visual field on day 7 (data not shown).

### Laser speckle Contrast Imaging Analysis

**Figure 4:**
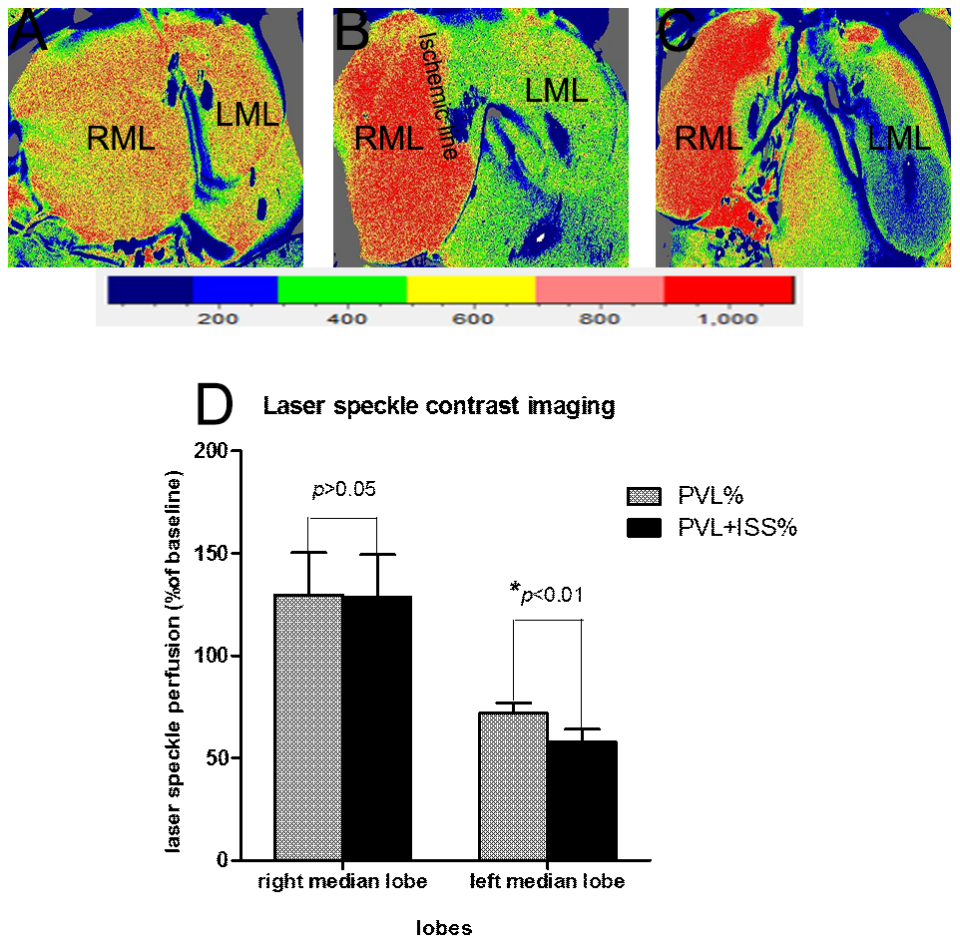
Laser speckle contrast imaging (LSCI). Typical hepatic flux perfusion images obtained from LSCI of the RML and the LML are shown: 5 min after laparotomy (**A**), 5 min after PVL (**B**) and 5 min after PVL+ISS (**C**). The microcirculation of the right median and left median lobes measured by LSCI after PVL and PVL+ISS was expressed as a percentage of the baseline value (**D**). There was a significant difference in the left median lobe before and after ISS (*p<0.01), but no significant difference was observed in the right median lobe (*p*>0.05).

LSCI was used to assess the liver microcirculation blood perfusion of the right and left median lobes before and after ISS. According to our records, the right median lobes had an increased microcirculation blood perfusion compared to baseline in both the PVL and PVL+ISS groups, but no significant difference was observed before and after ISS (129.72±20.38% versus 128.72±20.50%, p>0.05). However, the left median lobes had a significant difference in microcirculation flux before and after ISS (71.68±5.24% versus 58.07±5.91%, p<0.01), and the microcirculation flux was reduced after ISS **(** **Fig. 4** **)**.

### Hepatocellular Damage and Hepatic Synthetic Function

**Figure 5:**
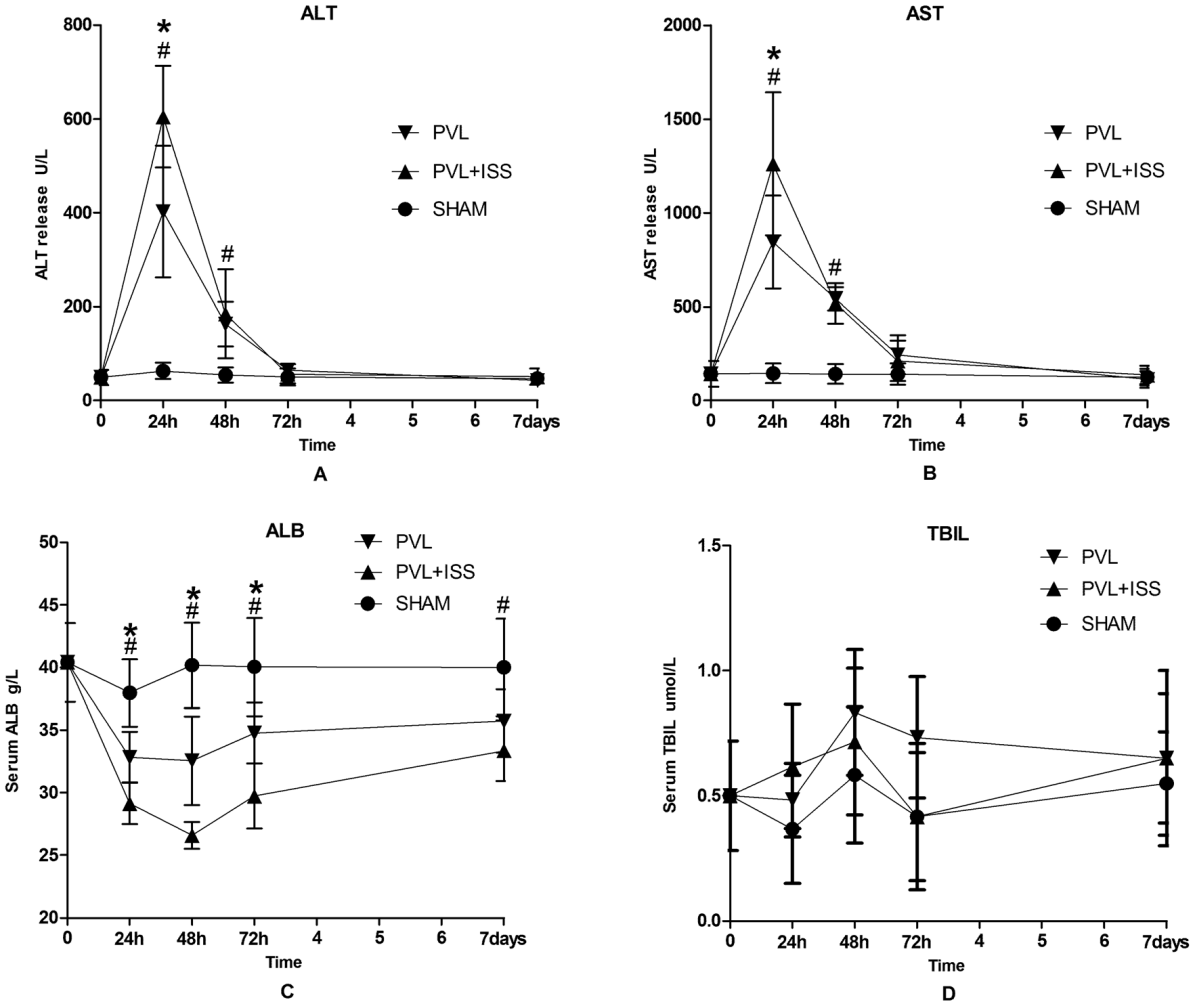
Biochemical indications after different surgical interventions at all time points. Serum levels of ALT (**A**), AST (**B**), ALB (**C**) and TBIL (**D**). Values are means±SD. There was a significant difference in ALT and AST release between PVL and PVL+ISS at 24 h (*p<0.05) after surgery and between PVL or PVL+ISS and SHAM at 24 and 48 h (^#^ *p*<0.01). Then, they returned to baseline values at 72 h. No significant difference was observed at 72 h and 7 days in all groups. Hepatocellular synthetic function evaluated by serum albumin level showed a prolonged dysfunction in both PVL and PVL+ISS groups (^#^ *P*<0.05 compared with the sham group at all time points). The serum albumin level of PVL+ISS was significantly different at 24, 48 and 72 h compared with the PVL group (*p<0.05). The serum levels of TBIL in all groups were not significantly different at any time point.

We measured serum aminotransferase (ALT and AST) levels as established markers of hepatocyte injury and performed hematoxylin-eosin staining on the left median lobes. Both PVL and PVL+ISS caused an obvious increase in ALT and AST levels compared with the SHAM group at 24 and 48 h. The ALT and AST levels in the PVL+ISS group were significantly different compared with the PVL group at 24 h, and they seemed to return to normal levels at 72 h. There were no significant differences at 72 h and 7 days among these three groups. TBIL levels in all groups were not significantly different at any time point **(** **Fig. 5** **)**.

**Figure 6:**
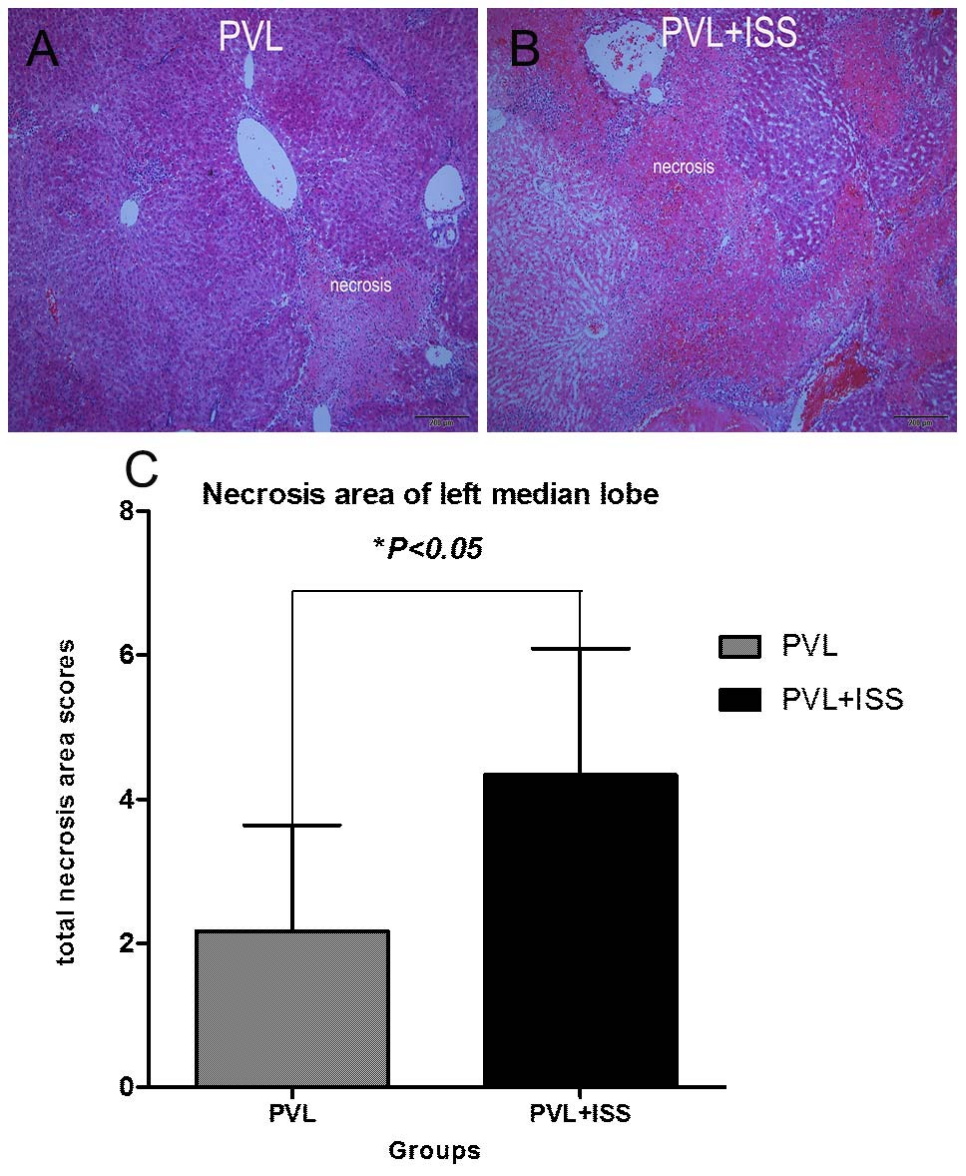
H-E staining of left median lobe at 24 h after surgery. Necrosis in PVL (**A**) and PVL+ISS (**B**) at 24 h after surgery. The total necrosis area scores of the left median lobes observed in 10 random visual fields (100×). Values are means±SD. There was a larger necrosis area in PVL+ISS than PVL. The necrosis area of the left median lobe in PVL+ISS was significantly different compared with that of the PVL group at 24 h (*p<0.05) (**C**).

Hematoxylin-eosin staining of the left median lobes after PVL and PVL+ISS revealed necrosis scores that were significantly larger after PVL+ISS than after PVL at 24 h after surgery (2.17±1.47 versus 4.33±1.75, *P*<0.01) **(** **Fig. 6** **)**. When the tissue was analyzed 7 days after the operation, hyaline degeneration had occurred in the areas of necrosis.

Significant differences in the serum albumin concentrations were observed in the PVL and PVL+ISS groups compared with the SHAM group at all time points. Serum albumin concentrations in the PVL+ISS group decreased considerably compared with the PVL group until the 72-h time point. The difference was significant **(** **Fig. 5** **)**. However, no difference was observed after 7 days (data not shown).

### The mRNA of Proinflammatory Cytokines Was Upregulated in the Regenerating Lobes

**Figure 7:**
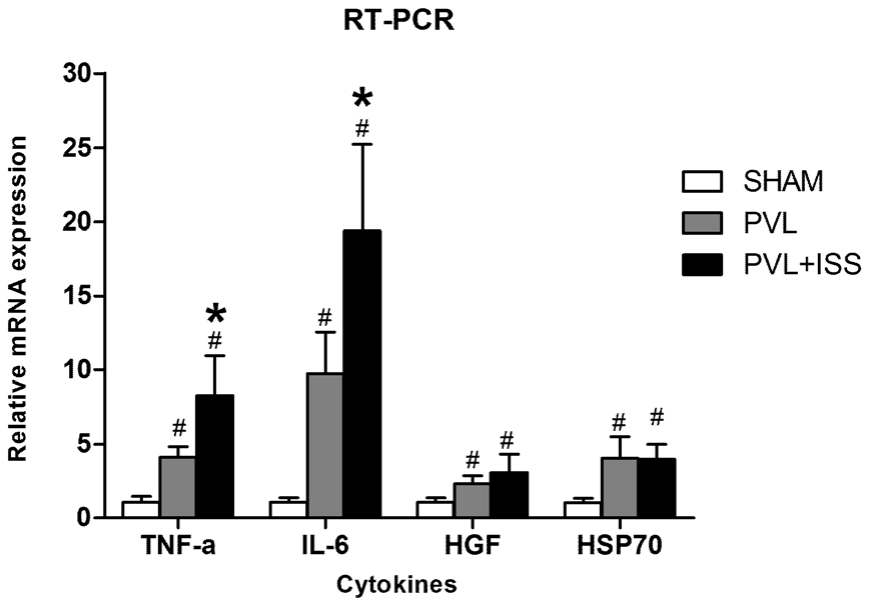
The mRNA expression of cytokines in regenerating liver tissue at 24 h after PVL and PVL+ISS. The relative mRNA expression levels of TNF-α, IL-6, HGF and HSP70 were determined by RT-PCR and given as fold inductions relative to the sham-operated livers. (^#^ *P*<0.01 compared with the sham group; *P<0.01 compared with the PVL group).

The expression of a large number of cytokine genes, which are believed to play critical roles in liver regeneration, was up-regulated in the regenerating liver lobes. To investigate a possible mechanism for the marked increased regeneration in the PVL+ISS group, we examined the mRNA levels of TNF-α, IL-6, HGF and HSP70 in the regenerating lobes 24 h post-surgery. As expected, these cytokines were highly upregulated in the regenerating lobes 24 h after PVL or PVL+ISS compared to sham-operated animals. In addition, we observed a significant increase in TNF-α and IL-6 mRNA in the PVL+ISS group compared with the PVL group. These data are consistent with the regenerative response assessed with HRR. There was no significant difference for the mRNA levels of HGF and HSP70 between the PVL and PVL+ISS groups, although HGF mRNA levels in the PVL+ISS group tended to be higher than those in the PVL group **(** **Fig. 7** **)**.

### Protein Levels of Proinflammatory Cytokines and HGF

**Figure 8:**
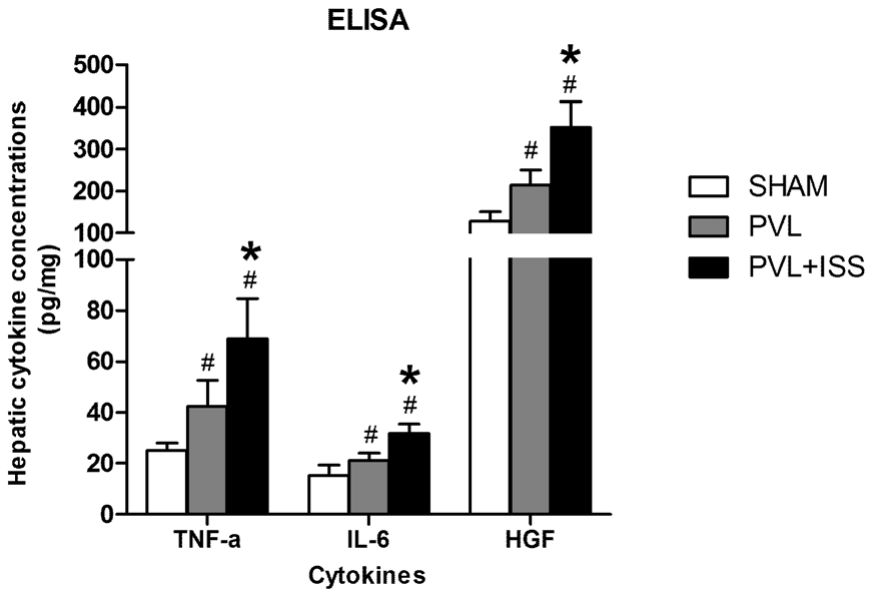
Protein levels of cytokines in regenerating liver tissue at 24 h after different surgical interventions. The protein levels of hepatic TNF-α, IL-6 and HGF in the PVL+ISS group had a significant difference compared with the PVL group. Both groups were significantly different from the SHAM group (^#^ *P*<0.05 compared with the SHAM group; *P<0.01 compared with the PVL group).

To further verify the role of the above-mentioned cytokines in liver regeneration, we also examined the hepatic TNF-α, IL-6 and HGF protein levels with ELISA at 24 h post-surgery. The data showed that hepatic TNF-α, IL-6 and HGF levels in the PVL+ISS group had a significant difference compared with the PVL group (*P*<0.05; data not shown). Both groups were significantly different from the SHAM group (*P*<0.05; data not shown) **(** **Fig. 8** **)**.

## Discussion

How to avoid postoperative liver failure because of insufficient remnant liver volume in hepatectomy has been a hot research issue for hepatobiliary surgeons in the past three decades [1]. The emergence of PVE and PVL no doubt improved this problem greatly. However, the main drawback of PVE or PVL is the long wait of approximately 4–16 weeks to obtain enough liver tissue before a two-stage hepatectomy, according to the literature [17], [18]. The long waiting period leads to some patients losing surgery opportunities due to metastasis or hepatic failure. In addition, there can still be an insufficient hypertrophy of the remnant liver in some patients despite a long wait period, which is also a shortcoming.

Schnitzbauer and his coworkers introduced a new technique, called “ALPPS”, which caused a heated discussion in the field of hepatobiliary surgery due to a surprising increase in the remnant liver volume in a short amount of time. However, there are still many issues requiring further investigation, such as its mechanism, indications and the related morbidity and mortality caused by ALPPS. To address these issues, we developed a model of PVL+ISS in animals.

In this study, we have successfully established a rat model of PVL+ISS. Although both the PVL and PVL+ISS groups were able to induce hypertrophy in the future remnant liver, PVL+ISS is more effective according to our data. The HRRs in the PVL+ISS group were significantly higher than that of the PVL group at 72 h and 7 days post-operation. Although the HRRs at 24 and 48 h were not significantly different, we speculated that the reason might be that hepatocyte proliferation was in the early stages at 24 h and that by 48 h, there had been a more increasing trend in the PVL+ISS compared with the PVL group, although no significant difference was observed. Ki-67 is commonly used as a molecular biological marker for regeneration. As expected, Ki-67 expression in the PVL+ISS group was stronger than that of the PVL group at 48 h and 72 h. We thought that the significant increase in the number of Ki-67-positive hepatocytes in the PVL+ISS group at 48 h was the basis for the higher hepatic regeneration rate in the PVL+ISS group at 72 h and 7 days post-operation, which was consistent with the HRR of the right median lobes. This further confirmed the reliability of our model.

In addition, the anatomy of the median liver lobe in rats is similar to humans, and the weight of the right median liver lobe induced hyperplasia in our study, accounting for approximately 26% of the total liver. This is comparable to humans in which it was thought that PVL or PVE was necessary prior to extensive hepatectomies when the remnant liver volume was less than 20% in the normal liver and less than 40% in the potentially diseased liver [19]–[21].

One explanation for the different HRRs in the right median lobes between PVL+ISS and PVL might be related to a different hepatic microcirculation perfusion before and after ISS. We compared hepatic microcirculation before and after ISS in identical rats to eliminate individual differences. The results showed a significant reduction in the microcirculation in the left median lobe after ISS. Meanwhile, the HE stain showed that the necrosis areas of the left median lobes were larger in the PVL+ISS group compared with the PVL group and that the serum ALT and AST levels, which represented the hepatocyte damage, were also high in the PVL+ISS group. These results seem to indicate that ISS leads to decreased microcirculation perfusion of the left median lobe, thereby causing injury or necrosis of liver cells. The serum ALB levels complied with the above results, which were lower than PVL alone until 72-h time point. The possible reasons included the larger necrosis areas of the left median lobes and decreased microcirculation and liver function was probably inhibited due to inflammation reaction and stress response caused by the in situ splitting. These factors potentially resulted in compensatory hypertrophy of the future remnant liver.

Does PVL+ISS cause changes in cytokines that are thought to be closely associated with liver regeneration? To further explore the molecular mechanisms, we examined the mRNA levels of TNF-α, IL-6, HGF and HSP70 in the regenerating lobes. The TNF-α and IL-6 mRNA levels were up-regulated significantly in the PVL+ISS group compared with the PVL only group. TNF-α and IL-6 play an important role in the initiation phase of liver regeneration. These two proinflammatory cytokines are produced by activated Kupffer cells of the liver and promote hepatocytes transitioning from the G0 phase to the G1 phase. Hepatocytes therefore become sensitive to some growth factors such as HGF and finally progress into DNA synthesis [22]–[24]. This suggests that ISS could promote the initiation of liver regeneration through upregulating these two proinflammatory cytokines. Contrary to our expectation, there were no differences in the HGF and HSP70 mRNA levels between the two groups, which represent a growth factor and stress factor, respectively.

To further confirm the above results, we also examined the protein levels of TNF-α, IL-6 and HGF in the regenerating liver tissue. The results of cytokines in the regenerating liver tissues showed that all of these cytokines were higher in the PVL+ISS group than the PVL group. This is consistent with their mRNA detection. These results suggested that the accelerated HRR in PVL+ISS was associated with some cytokine upregulation, especially TNF-α and IL-6. And we speculated the increased cytokines in our experiment might have some connection with proinflammatory and stress response caused by the ISS and necrosis of hepatocytes because of decreased microcirculation in LML.

In conclusion, we successfully established a PVL+ISS model in rats and demonstrated the accelerated hepatic regeneration in PVL+ISS compared with PVL only. The mechanisms for liver regeneration include the reduced blood supply to the left median lobe and upregulated cytokines in the regenerating lobes.

There are some limitations in this study. The difference in the HRR between PVL and PVL+ISS is less than that which would occurs in humans due to the different liver anatomies of rats and humans. However, our model provides a basis for further studies of ALPPS. Moreover, it will be helpful to explore the reasons for various complications that occur in ALPPS. If we could reduce the incidence of complications effectively, ALPPS will play a crucial role in patients who lose surgery opportunities because of their insufficient remnant liver volumes.

## References

[1] Clavien PA, Petrowsky H, DeOliveira ML, Graf R (2007) Strategies for safer liver surgery and partial liver transplantation. N Engl J Med 356: 1545–1559. 17429086 10.1056/NEJMra065156

[2] Shirabe K, Shimada M, Gion T, Hasegawa H, Takenaka K, et al (1999) Postoperative liver failure after major hepatic resection for hepatocellular carcinoma in the modern era with special reference to remnant liver volume. J Am Coll Surg 188: 304–309. 10065820 10.1016/s1072-7515(98)00301-9

[3] Makuuchi M, Thai BL, Takayasu K, Takayama T, Kosuge T, et al (1990) Preoperative portal embolization to increase safety of major hepatectomy for hilar bile duct carcinoma: a preliminary report. Surgery 107: 521–527. 2333592

[4] Ribero D, Abdalla EK, Madoff DC, Donadon M, Loyer EM, et al (2007) Portal vein embolization before major hepatectomy and its effects on regeneration, resectability and outcome. British Journal of Surgery 94: 1386–1394. 17583900 10.1002/bjs.5836

[5] Liu H, Zhu S (2009) Present status and future perspectives of preoperative portal vein embolization. The American Journal of Surgery 197: 686–690. 19249737 10.1016/j.amjsurg.2008.04.022

[6] Abulkhir A, Limongelli P, Healey AJ, Damrah O, Tait P, et al (2008) Preoperative Portal Vein Embolization for Major Liver Resection. Annals of Surgery 247: 49–57. 18156923 10.1097/SLA.0b013e31815f6e5b

[7] Schnitzbauer AA, Lang SA, Goessmann H, Nadalin S, Baumgart J, et al (2012) Right Portal Vein Ligation Combined With In Situ Splitting Induces Rapid Left Lateral Liver Lobe Hypertrophy Enabling 2-Staged Extended Right Hepatic Resection in Small-for-Size Settings. Annals of Surgery 255: 405–414. 22330038 10.1097/SLA.0b013e31824856f5

[8] de Santibañes E, Clavien P-A (2012) Playing Play-Doh to Prevent Postoperative Liver Failure. Annals of Surgery 255: 415–417. 22330039 10.1097/SLA.0b013e318248577d

[9] Tschuor C, Croome KP, Sergeant G, Cano V, Schadde E, et al (2013) Salvage parenchymal liver transection for patients with insufficient volume increase after portal vein occlusion – An extension of the ALPPS approach. European Journal of Surgical Oncology (EJSO) 39: 1230–1235. 23994139 10.1016/j.ejso.2013.08.009

[10] Neumann UP, Dejong CH (2013) Split decision. Br J Surg 100: 310–312. 23300069 10.1002/bjs.9050

[11] Machado MA, Makdissi FF, Surjan RC (2013) ALPPS Procedure with the Use of Pneumoperitoneum. Annals of Surgical Oncology 20: 1491–1493. 23468045 10.1245/s10434-013-2920-y

[12] Kilkenny C, Browne WJ, Cuthill IC, Emerson M, Altman DG (2010) Improving bioscience research reporting: the ARRIVE guidelines for reporting animal research. PLoS Biol 8: e1000412. 20613859 10.1371/journal.pbio.1000412 PMC2893951

[13] McGuire PG, Howdieshell TR (2010) The Importance of Engraftment in Flap Revascularization: Confirmation by Laser Speckle Perfusion Imaging. Journal of Surgical Research 164: e201–e212. 20863524 10.1016/j.jss.2010.07.059

[14] Sturesson C, Milstein DMJ, Post ICJH, Maas AM, van Gulik TM (2013) Laser speckle contrast imaging for assessment of liver microcirculation. Microvascular Research 87: 34–40. 23403398 10.1016/j.mvr.2013.01.004

[15] Veteläinen R, Dinant S, van Vliet A, van Gulik TM (2006) Portal Vein Ligation Is as Effective as Sequential Portal Vein and Hepatic Artery Ligation in Inducing Contralateral Liver Hypertrophy in a Rat Model. Journal of Vascular and Interventional Radiology 17: 1181–1188. 16868172 10.1097/01.RVI.0000228460.48294.2E

[16] Livak KJ, Schmittgen TD (2001) Analysis of Relative Gene Expression Data Using Real-Time Quantitative PCR and the 2−ΔΔCT Method. Methods 25: 402–408. 11846609 10.1006/meth.2001.1262

[17] Seyama Y, Makuuchi M (2007) Current surgical treatment for bile duct cancer. World J Gastroenterol 13: 1505–1515. 17461441 10.3748/wjg.v13.i10.1505 PMC4146891

[18] Jaeck D, Oussoultzoglou E, Rosso E, Greget M, Weber J-C, et al (2004) A Two-Stage Hepatectomy Procedure Combined With Portal Vein Embolization to Achieve Curative Resection for Initially Unresectable Multiple and Bilobar Colorectal Liver Metastases. Annals of Surgery 240: 1037–1051. 15570209 10.1097/01.sla.0000145965.86383.89 PMC1356519

[19] Kishi Y, Abdalla EK, Chun YS, Zorzi D, Madoff DC, et al (2009) Three Hundred and One Consecutive Extended Right Hepatectomies. Transactions of the Meeting of the American Surgical Association 127: 171–179.

[20] Ribero D, Curley SA, Imamura H, Madoff DC, Nagorney DM, et al (2008) Selection for Resection of Hepatocellular Carcinoma and Surgical Strategy: Indications for Resection, Evaluation of Liver Function, Portal Vein Embolization, and Resection. Annals of Surgical Oncology 15: 986–992. 18236112 10.1245/s10434-007-9731-y

[21] Abdalla EK (2010) Portal vein embolization (prior to major hepatectomy) effects on regeneration, resectability, and outcome. Journal of Surgical Oncology 102: 960–967. 21165999 10.1002/jso.21654

[22] Fujiyoshi M, Ozaki M (2011) Molecular mechanisms of liver regeneration and protection for treatment of liver dysfunction and diseases. J Hepatobiliary Pancreat Sci 18: 13–22. 20607568 10.1007/s00534-010-0304-2

[23] Pahlavan PS, Feldmann RE Jr, Zavos C, Kountouras J (2006) Prometheus’ challenge: molecular, cellular and systemic aspects of liver regeneration. J Surg Res 134: 238–251. 16458925 10.1016/j.jss.2005.12.011

[24] Taub R (2004) Liver regeneration: from myth to mechanism. Nat Rev Mol Cell Biol 5: 836–847. 15459664 10.1038/nrm1489

